# The Involvement of WDHD1 in the Occurrence of Esophageal Cancer as a Downstream Target of PI3K/AKT Pathway

**DOI:** 10.1155/2022/5871188

**Published:** 2022-04-05

**Authors:** Qingying Xian, Danxia Zhu

**Affiliations:** Department of Oncology, The Third Affiliated Hospital of Soochow University, Changzhou, Jiangsu Province, China

## Abstract

Esophageal cancer is one of the most common malignant tumors in the world, which is characterized by high incidence, strong invasiveness, high mortality, and poor prognosis. At present, the therapies include surgery, endoscopic resection, radiotherapy and chemotherapy, targeted therapy, and immunotherapy. The five-year survival rate of esophageal cancer has not been significantly improved, although the medical level has been continuously improved and the management and application of different therapies have been improved day by day. At present, an abnormal gene expression is still regarded as an important factor in the occurrence and development of esophageal cancer. WD repeat and HMG-box DNA binding protein 1(*WDHD1*), as a key gene, plays an important role in the occurrence of esophageal cancer. It is known that the protein encoded by *WDHD1* is the downstream target of the PI3K/AKT pathway. When PI3Ks is activated by extracellular signals, PI(4,5)P2 on the inner side of the plasma membrane will be converted into PI(3,4,5)P3. Then, PI(3,4,5)P3 can be converted into PI(3,4)P2,PI(4)P and PI(3)P by dephosphorylation of some regulatory factors. PI(3,4,5)P3 recruited AKT to the plasma membrane and combined with its pH domain, resulting in conformational change of AKT. Subsequently, AKT was completely activated by PDK1 and PDK2 and begins to move to the cytoplasm and nucleus. In this process, AKT continuously phosphorylates downstream substrates. WDHD1, as a downstream target of AKT, is also phosphorylated and induces DNA replication. Besides the abnormal regulation of cells by other downstream targets of AKT, it also becomes a potential pathway that may eventually lead to the occurrence of esophageal cancer.

## 1. Introduction

**Figure 1:**
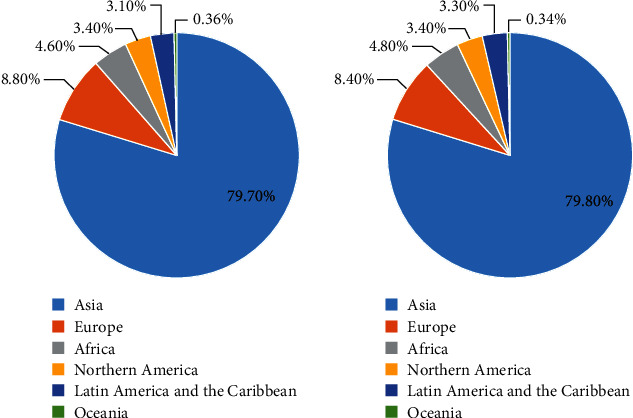
In 2020, the number of new cases of esophageal cancer worldwide was 604,100 (a), and the number of deaths from esophageal cancer worldwide was 544,076 (b) (source: GLOBOCAN 2020, International Agency for Research on Cancer, available online: http://globocan.iarc.fr).

Esophageal cancer is one of the most common malignant tumors in the world [1], ranking eighth in the incidence of all tumors [2], and it is also a highly invasive gastrointestinal tumor [3], ranking sixth in cancer-related deaths worldwide [4]. In the United States, there are only 17,000 newly diagnosed cases of esophageal cancer each year, but the estimated mortality rate exceeds 80%; and in China, about 250,000 people die of this disease each year [2, 5], and the incidence rate of esophageal cancer in China accounts for more than 70% of the world [1]. According to statistics, there were 604,100 new cases and 54,076 death cases of esophageal cancer worldwide in 2020 (Figure 1). The most common subtypes of esophageal cancer are esophageal squamous cell carcinoma (ESCC) and esophageal adenocarcinoma (EAC), accounting for more than 95% of esophageal cancer, while the rare subtypes of esophageal cancer account for a small proportion, including lymphoma, melanoma, carcinoid and sarcoma, basal cell carcinoma, transitional cell carcinoma, and other unspecified cancers [6, 7]. There are geographical differences between the incidences of ESCC and EAC: the incidences of ESCC are higher in East Asia, East Africa, Southern Africa, and Southern Europe, while EAC is more common in parts of Europe and North America [3]. Squamous cell carcinoma originates from the squamous epithelial cells of the esophagus and usually occurs in the upper and middle parts of the esophagus. Important risk factors of ESCC include smoking, drinking alcohol, achalasia, low socioeconomic status, hot drinks, micronutrient deficiency, low intake of fruits and vegetables, low socioeconomic status, corrosive damage to the esophagus caused by intake of lye, radiation therapy, nitrosamines, polycyclic hydrocarbons, and acetaldehyde (Short et al., 2017; Smyth et al., 2017; Moehler et al., 2018; Naveed and Kubiliun, 2018; Chang and Worrell, 2020; Uhlenhopp et al., 2020). Adenocarcinoma originated from glandular cells near the stomach, which is considered to be closely related to acid exposure in the lower esophagus. Adenocarcinoma usually occurs in the distal esophagus, the important risk factors including male, chronic gastroesophageal reflux disease, obesity, alcohol, tobacco, Barrett's esophagus, large intake of red meat, and insufficient intake of fruits and vegetables [6–11]. Although the management, diagnosis, and treatment of esophageal cancer have been improved in recent years, the overall effect is still not satisfactory: the overall 5-year survival rate is about 10%, while the 5-year survival rate after esophagectomy is about 15-40% [12]. Surgery at the junction of esophagus or stomach-esophagus greatly affects the quality of life of patients. Because of the decrease of stomach volume, there will be early reflux of saturated stomach content, and nausea and vomiting will often occur. When upper digestive tract resection is combined with vagotomy, gastric retention and frequent dilatation will occur, and postoperative complications such as anastomotic leakage may appear [10, 13]. At present, the standard treatment for esophageal cancer is limited to surgery, endoscopic resection, and chemoradiotherapy. About 50% patients diagnosed with esophageal cancer have obvious metastatic diseases. These patients are mainly treated with palliative chemotherapy, but early local esophageal cancer will also have metastasis. Therefore, most patients with esophageal cancer need systemic chemotherapy, such as 5-fluorouracil, cisplatin, taxane, irinotecan, anthracycline, and other chemotherapeutic drugs. However, even if targeted therapy is added on the basis of chemotherapy regimen, the 5-year survival rate of esophageal cancer is still low, about 15%-25% [14, 15]. With the continuous improvement of medical level, an immune checkpoint inhibitor has become a new treatment option for esophageal cancer, and other immunogenic methods such as polypeptide vaccine, adoptive cell therapy, and oncolytic virus are being developed, which is expected to bring long-term benefits for esophageal cancer patients [16].

**Figure 2:**
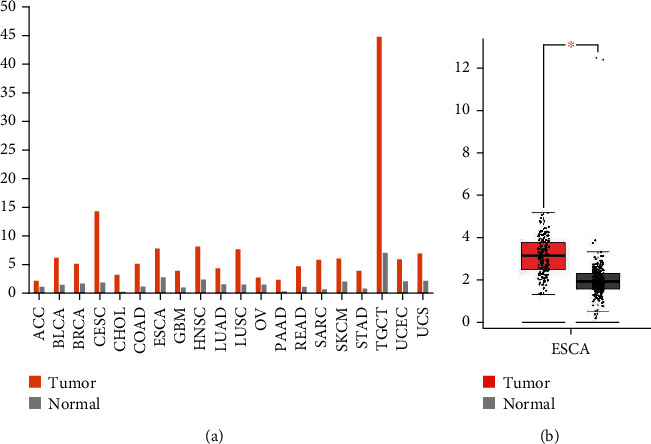
(a) Gene expression profiles of *WDHD1* in different tumor tissues and paired normal tissues, and the *Y* axis represents the median expression of *WDHD1* in some tumor types or normal tissues. (b) Difference of expression levels of *WDHD1* in esophageal cancer and paired normal tissues; the *Y* axis represents the relative expression amount of WDHD1, ^∗^*P* < 0.05. ACC: adrenocortical carcinoma; BLCA: bladder urothelial carcinoma; BRCA: breast invasive carcinoma; CESC: cervical squamous cell carcinoma and endocervical adenocarcinoma; CHOL: cholangio carcinoma; COAD: colon adenocarcinoma; ESCA: esophageal carcinoma; GBM: glioblastoma multiforme; HNSC: head and neck squamous cell carcinoma; LUAD: lung adenocarcinoma; OV: ovarian serous cystadenocarcinoma; PAAD: pancreatic adenocarcinoma; READ: rectum adenocarcinoma; SARC: sarcoma; SKCM: skin cutaneous melanoma; STAD: stomach adenocarcinoma; TGCT: testicular germ cell tumors; UCEC: uterine corpus endometrial carcinoma; UCS: uterine carcinosarcoma.

The pathogenesis of esophageal cancer has not been thoroughly studied, but abnormal gene expression is still considered to be an important factor for the occurrence of esophageal cancer, and some studies have pointed out that WD repeat and HMG-box DNA binding protein 1 (*WDHD1*) is the hub gene in the occurrence of esophageal cancer [17]. At present, multiple studies have confirmed that WDHD1 is closely related to different tumors (Table 1). And depletion of WDHD1 may result in cells being more sensitive to chemotherapeutic agents such as etoposide and PARP inhibitors for clinical treatment of patients with breast, ovarian, and prostate cancer [18]. We searched the GEPIA database for the expression of *WDHD1* and found that in most tumors, the expression of *WDHD1* in tumor tissues was higher than that in normal tissues (Figure 2(a)). Then, we analyzed the expression levels of WDHD 1 in 182 cases of esophageal cancer and 286 cases of normal tissues in the GEPIA database. We found that the expression level of WDHD 1 in esophageal cancer tissues was higher than that in normal tissues, and the difference was statistically significant (Figure 2(b)). However, the effect of WDHD1 on esophageal cancer is rarely reported. This manuscript reviews the relationship between WDHD1 and esophageal cancer.

## 2. WDHD1

WDHD1 refers to WD repeat and HMG-box DNA binding protein 1, also known as acidic nucleoplasmic DNA binding protein 1 (AND-1), and human chromosome transmission fidelity factor 4 (hCTF4). It is a kind of DNA binding protein in the nucleus and cytoplasm, which controls mitosis, DNA damage, and repair. Meanwhile, WDHD1 is also a ubiquitin ligase, which is related to tumor development and malignant phenotype [17, 19, 30].WDHD1 is a human homologue of CTF4 (chromosome transmission fidelity factor 4) in Saccharomyces cerevisiae [31]. WDHD1 is similar to the sequence of CTF4 (722 of 1129 aligned residues have 21% homology and 36% similarity). It is precisely for this reason that WDHD1 may have some or all functions of its yeast counterpart in maintaining the stability of genome and building the cohesion of sister chromatids [32]. WDHD1 is mainly involved in the regulation of the cell DNA replication and division cycle [24, 33]. DNA replication in eukaryotic cells is performed by a multiprotein complex called replica, which consists of helicase, polymerase, and linker molecules, of which WDHD1 is the key linker molecule for DNA replication [34]. WDHD1 is located near the DNA replication site and plays an important role in the initial process of DNA replication [35, 36]. More scholars claim that WDHD1 plays a role in replication induced by viral oncogenes, and it is related to cell cycle checkpoint control, epithelial-mesenchymal transition, tumor growth, and metastasis [37].

WDHD1 is an acidic cytoplasmic DNA binding protein of 126 kilodaltons (kDa). WDHD1 is relatively conserved in evolution and has homologues in most eukaryotes, containing 1129 amino acids [38, 39], mainly including the amino-terminal WD 40 repeat domain, Sep B domain, and a carboxyl-terminal HMG domain. WD 40 repeat domain forms a circular *β*-spiral structure, and it not only mediates the interaction between proteins but also functions as a linker/modulator in signal transduction, pre-mRNA processing, and cytoskeleton assembly; SepB is the most conservative domain in WDHD1; HMG domain can participate in DNA binding, which is a kind of nonhistone component, and mainly participates in chromatin assembly, transcription, replication, and regulation of nuclear protein complex assembly [21, 33, 38, 40–44]. WDHD1 generally exists as a trimer, with the N-terminal WD 40 repeat domain and the extended C terminal region across the HMG box being flexibly oriented in the trimer structure, and the SepB domain being the trimerization region of the WDHD1 [41]. SepB domain consists of a six-bladed propeller and a C- terminal five-helical bundle [32]. The WD 40 domain, SepB domain, and HMG domain are connected by two flexible loops, which are composed of 120 and 170 amino acids, respectively. The HMG domain at the C-terminal of WDHD1 is a DNA binding module that recognizes various DNA conformations. Some scholars claim that WDHD1 is the hub in human cells, which can realize cell function by connecting proteins containing multiple WDHD1 interacting peptides [34].

## 3. PI3K-AKT Pathway and WDHD1 in Esophageal Cancer (Figure 3)

As one of the important pathways related to cell survival, proliferation, autophagy, and apoptosis [45], the PI3K/AKT pathway has been studied extensively in normal and malignant tumor cells [46]. Deficiency of the PI3K/AKT signal pathway leads to abnormal signal activity, which leads to various diseases, such as diabetes, cardiovascular, neurological, and hematological diseases [47, 48], and the upregulation of the PI3K/AKT signal pathway is also considered one of the characteristics of cancer [49]. WDHD1 is closely related to the occurrence of many cancers. Currently, studies have proved that *WDHD1* is the central gene for the occurrence and development of esophageal cancer [17].

### 3.1. PI3Ks

PI3K (phosphatidylinositol 3-kinase) is a family of intracellular heterodimeric lipid kinases, which share common core motifs, including C2 domain, helix domain, and catalytic domain. PI3K has the ability to phosphorylate phosphatidylinositol 3′-OH group on cell membrane and responds to environmental signals such as nutrition and growth factors and plays an important regulatory role in cell growth, proliferation, survival, differentiation, metabolism, apoptosis, movement, genome stability, protein synthesis, and angiogenesis, most of which are the basic factors of tumorigenesis [50–54]. There are eight species of PI3K in mammals. According to different coding genes, structure, and substrates, PI3K can be divided into three categories: Class I, Class II, and Class III (see Table 2 for the classification and function). Class I PI3K consists of IA and IB, both of which are activated by cell surface receptors. PI3K IA is a heterodimer composed of p85 regulatory subunit and p110 catalytic subunit; it is obviously related to cancer [46, 52, 55]. In addition to catalytic activity, class I PI3K catalytic subunit also has the function of protein scaffold, which acts as adaptor proteins in the assembly of protein-protein complex and regulates a series of biological functions independent of PI3K catalytic activity [56].

Class II PI3K is a monomeric catalytic isomer that contains only one catalytic subunit similar to p110 but no regulatory subunit. Its catalytic subunit includes RAS binding domain (RBD), helix domain, and catalytic domain [57]. And class II PI3K is associated with membrane transport and internalization of receptors [58, 59]. Class III PI3K is composed of catalytic subunit vacuole protein sorting 34 (Vps34) encoded by PIK3C3 and regulatory subunit vacuole protein sorting 15 (Vps15) encoded by PIK3R4 [60]. Vps34 and Vps15 play an important role in regulating autophagy [61]. In this review, when we summarize the relationship between the PI3K/AKT pathway and esophageal cancer, we focus on the relationship between class I PI3K and esophageal cancer.

### 3.2. AKT

A downstream molecules of PI3K is the 57 kDa serine/threonine kinase AKT, also known as protein kinase B (PKB), which is one of the most active kinases in human cancer and is conserved in advanced eukaryotes. AKT can influence cell cycle, transcription, translation, apoptosis, differentiation, survival, metabolism, and cytoskeleton recombination. In eukaryotes, there are three isomers with high homology: AKT1, AKT2, and AKT3, are coded by *PKBα*, *PKBβ*, and *PKBγ*, respectively. These three subtypes have different structures and functions. One subtype of AKT can not completely replace another subtype [46, 49, 52, 55].

Three subtypes of AKT have 85% amino acid sequence homology and very similar three-dimensional structures. AKT is composed of three different functional domains, including N-terminal pH domain (~110 amino acids) which mediates lipid-protein and protein-protein interactions, a catalytic domain (~260 amino acids, this region is specific to the serine or threonine residues of the substrate protein) which contains threonine phosphorylation site, and C-terminal regulatory domain (~70 amino acids, this region is a hydrophobic and proline-rich domain) which has serine phosphorylation sites. Among them, threonine and serine sites are necessary for PI3K to activate downstream-dependent AKT. Activated AKT is transferred into the nucleus and phosphorylates its downstream substrate [46, 48, 51, 67, 68].

### 3.3. WDHD1 and PI3K/AKT Pathways in Esophageal Cancer

The activation of class I PI3K leads to the release of p110, which is recruited to plasma membrane for activation, and then phosphatidylinositol-4,5-diphosphate (PI(4,5)P2) inside plasma membrane is converted into phosphatidylinositol-3,4,5-triphosphate (PI [3,4,5] P3). PIP3, as the second messenger, can bind and recruit the lipid binding domain (mostly pH domain) of the downstream target to the cell membrane. In addition, class I PI3K can also activate a wide range of downstream signals by directly binding to the pH domain, thereby locating on the cell membrane and activating the cell growth and cell survival pathways. Phosphatase and tensin homologue deleted on Chromosome 10 (PTEN) regulates this pathway by targeting PI [3,4,5]P3 to the phosphate at the third position of the inositol ring and dephosphorylating PI [3,4,5] P3 to PI [3,4]P2, and P I[4]P. PTEN, as the main negative regulator of the PI3K pathway, blocks the activation of downstream kinases. It has also been reported that the PI3K pathway is activated by downregulating or deleting PTEN. The decrease of PTEN level or PTEN activity leads to the accumulation of PI(3,4,5)P3, which is also related to the activation of the oncogene AKT. Therefore, the existence and activation of PTEN on the cell membrane is crucial to ensure the controlled transduction of PI3K signal [45, 46, 51–53, 55, 62, 69–72]. Moreover, p85*α*-mediated mechanism that promotes the generation of PI(3,4,5)P3 can also initiate a protective response that terminates the function of PI(3,4,5)P3 by promoting the supplementation and participation of PTEN. PTEN exists in the form of nonphosphorylated dimer, which makes PI(3,4,5)P3 dephosphorylated. The fine balance of homologues (PTEN : PTEN and p85*α* : p85*α*) and heterodimers (p85*α* : p110 and p85*α* : PTEN) also forms the PTEN-related complex (PAC) [71], which finely regulates the normal activation of the PI3K pathway. It should be noted that PI(3,4,5)P3 is only produced by class I PI3K, while different PIP2 and PIP are produced by more than one variety of PI3K. PI(3,4,5)P3 is dephosphorylated via SHIP1(SH2 domain-containing inositol 5′-phosphatase 1) and SHIP2 (SH2 domain-containing inositol 5′-Phosphatase 2)to PI(3,4)P2, which is then dephosphorylated by INPP4A (inositol polyphosphate 4-phosphatase type I) and INPP4B (inositol polyphosphate 4-phosphatase type II) to produce PI(3)P [56, 61]. Therefore, PTEN, PAC, SHIP1, SHIP2, INPP4A, and INPP4B are negative regulators of the PI3K signal pathway, and their mutations can lead to the accumulation of P I[3,4,5]P3 and PIP2, which leads to the long-term activation of the PI3K signal pathway. PI3K is activated by many genes, which leads to AKT binding to cell membrane in the PI3K/AKT signal transduction pathway with the participation of phosphoinositide-dependent kinase. The phosphorylation of threonine and serine promotes the transfer of AKT from cytoplasm to the nucleus and further mediates the biological effects of enzymes, including participating in cell proliferation, apoptosis inhibition, cell migration, vesicle transportation, and cell carcinogenesis [59]. AKT can be activated by many external signals, such as growth factors, cytokines, oncogenes, and cell stress conditions. AKT is localized in the cytoplasm before activation, driven by PI(3,4,5)P3 and recruited to the plasma membrane for activation. Interaction with PI(3,4,5)P3 leads to the conformational changes of AKT. AKT binds to upstream kinases through its pH domain, such as phosphoinositide-dependent kinase 1 (PDK1), and then PDK1 phosphorylates the kinase domain of AKT (threonine of 308/309/305 in AKT1/2/3). The complete activation of AKT requires phosphoinositide-dependent kinase 2 (PDK2) to phosphorylate the carboxyl terminal hydrophobic of AKT (serine of 473/474/472 in AKT1/2/3). Once activated, AKT is transferred to the cytoplasm and nucleus, during which many downstream targets are phosphorylated, activated, or inhibited. AKT can phosphorylate a variety of substrates to regulate cellular processes, including proliferation, survival, movement, angiogenesis, and glucose metabolism homeostasis [46–49, 52, 55, 56, 63, 66, 67, 72–75]. AKT is a kind of phosphorylated protein, and a large number of hydrophobic mammalian proteins containing the AKT common phosphorylation site RXRXXS/T-B (X can be any amino acid, and B is a huge hydrophobic amino acid) are substrates of AKT, but substrate specificity of AKT also depends on sequences of downstream protein other than the RXRXXS/T-B. AKT promotes the survival and growth of cells and biosynthesis of various anabolisms by directly or indirectly phosphorylating these target proteins [67, 68, 76]. Inactivation of AKT is realized by dephosphorylation of threonine residues by protein phosphatase 2 (PP2A) and serine residues by pH domain leucine-rich repeat protein phosphatase (PHLPP) [55, 60]. PHLPP, with the phosphate group removed from AKT serine site [77], is composed of two subtypes, namely, PHLPP1 and PHLPP2. PHLPP1 specifically targeted AKT2, while PHLPP2 preferentially inactivates AKT3 [78]. AKT mutations rarely occur, but amplification and overexpression of AKT gene (the most common one is *AKT1*) can be found in various cancers, including gastric cancer, breast cancer, colon cancer, esophageal cancer, ovarian cancer, pancreatic cancer, thyroid cancer, and glioblastoma [67].

**Figure 4:**
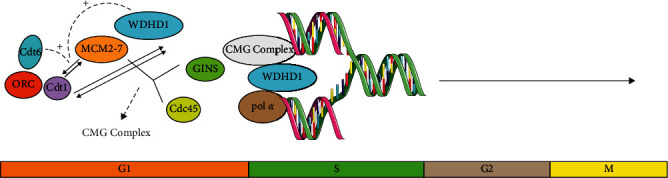
WDHD1 and DNA replication.

The PI3K/AKT pathway can promote tumor progression by stimulating cell proliferation and angiogenesis and inhibiting apoptosis in different types of tumors [79]. Up to now, many researches have proved that the PI3K/AKT pathway is abnormally activated in esophageal cancer [80–82]. Scholars have studied 49 pairs of human esophageal tumors and normal tissues and found that, compared with the corresponding normal tissues, AKT in most of esophageal tumors (75.5%) are structurally activated. Using specific inhibitors of the PI3K/AKT pathway can significantly reduce the expression of antiapoptosis protein Bcl-xl and induce caspase-3- (caspase-3-) dependent apoptosis, thus inhibiting cell proliferation and tumor growth in vivo [83]. Wang et al. studied the relationship between marmesin and esophageal cancer by targeting the PI3K/AKT pathway, they found that Marmesin exerted anticancer activity in esophageal cancer cells by inhibiting PI3K/AKT pathway, and speculated that PIK3CA was a key oncogene related to the activation of the PI3K/AKT pathway [79]. To sum up, we have fully explored the activation process of PI3K/AKT, and we want to put forward the viewpoint that WDHD1, as the downstream target of PI3K/AKT pathway, is involved in the occurrence of human esophageal cancer. Some scholars identified the central genes related to the pathogenesis of esophageal cancer through weighted gene coexpression network analysis (WGCNA), including WDHD1, whose expression in esophageal cancer tissue increased significantly [17]. Sato et al. [31] found a common phosphorylation site (R-X-R-X-X-X-S374) related to AKT kinase in WDHD 1 protein and detected a positive band that WDHD1 might be phosphorylated by endogenous AKT. Subsequently, the researchers used the labeled WDHD1 immunoprecipitation as a substrate and the recombinant human AKT1 protein as a kinase. In kinase analysis of PAS antibody, immunoblotting also proved that AKT1 phosphorylated WDHD1 directly. A series of experiments have proved that WDHD1 was the downstream target of the PI3K/AKT pathway. The study also showed that the survival time of WDHD1 gene-positive esophageal cancer patients was shorter than that of WDHD1 gene-negative esophageal cancer patients through tissue chip experiments. The research of WDHD mainly focuses on DNA replication, and the close relationship between WDHD and DNA replication has been confirmed [33, 37, 38, 40]. The initial process of DNA replication includes two steps, namely, the assembly and activation of the prereplication complex (Pre-RC); WDHD1 plays a role in both processes above [37]. The WDHD1 of human cells is assembled to chromatin in late mitosis and early G1 phase and then assembled into Pre-RC. Pre-RC starts with the assembly of 6-subunit origin recognition complex (ORC) at the beginning of replication and then promotes the recruitment of replication factors Cdt1 and Cdc6. MCM2-7 complex, a DNA helicase, continuing to be recruited by ORC, Cdt1, and Cdc6 to the starting point. Cdt1 interacted with MCM2-7, Cdc6 enhanced the interaction between Cdt1 and MCM2-7, two proteins that promote the assembly of MCM2-7 at the initial site. And WDHD1 formed a complex with MCM2-7. The downregulation of WDHD1 significantly inhibited the chromatin loading of MCM2-7 in the anaphase of mitosis and G1. In addition, we found that human WDHD1 interacted with Cdt1, and the lack of WDHD1 inhibits the interaction of Cdt1 and MCM2-7 in the G1 phase, so WDHD 1 controls the assembly of MCM2-7 at the beginning of replication [38]. In order to activate Pre-RC and form replicative helicase, Cdc45 (cell division cycle 45) and GINS (Go, Ichi, Nii and San) were recruited to the Pre-RC and interacted with the MCM2-7 to form the CMG complex [84]. WDHD1 coordinates *α*-melting and polymerase activity through interaction with the DNA polymerase *α* and CMG helicase complexes. WDHD1 were recruited to replication fork as a scaffold for various proteins, thus participating in DNA replication [35, 85]. WDHD1 is necessary to stabilize DNA polymerase *α* in human cells, and it is also necessary in the late S-phase [36]. WDHD1 is a hub in human cells, which functions by connecting proteins containing several interacting peptides of WDHD1, and it is the key connecting molecule for DNA replication [34].WDHD1, a replication factor, is necessary for the effective replication of DNA in normal cells and tumor cells [86]. Studies have shown that WDHD1 plays a major role in cell proliferation during mouse embryonic development [87]. But for tumor cells, WDHD1 is necessary for DNA replication and effective progression and homologous recombination repair in S phase, and the deletion of WDHD1 increases the accumulation of DNA damage, delays the progression of S phase, halts the cell cycle in late S phase to G2 phase, leads to the accumulation of cells in late S phase and/or G2 phase, and induces the death of cancer cells [35]. Experiments by Sato et al. also proved that WDHD1 knockout delayed the entry and progress of the cell cycle of S phase and led to the death of cancer cells immediately after cell division [31]. In addition, WDHD1 has the remarkable ability to regulate the stability of general control nonderepressible (Gcn5) after interacting with Gcn5, while Gnc5 is related to gene transcription mediated by oncogene c-Myc and E2F, which has been proved to be related to oncogene regulation [44]. Therefore, we speculate that WDHD1 is a downstream target of the PI3K/AKT pathway, and it will become a potential tumor promoter by influencing DNA replication in the cell cycle (Figure 4).

At present, the two most extensive activation mechanisms of PI3K/AKT found in human cancer are triggered by receptor tyrosine kinases (RTK) and somatic mutation of specific components of signaling pathways, which are related to tumor growth, angiogenesis, and survival. The mechanism of PI3K pathway stimulation may have a negative impact on treatment methods, so inhibiting PI3K may have beneficial clinical effects [52]. There are many downstream targets in the PI3K/AKT signal transduction pathways. For example, the activation of AKT can inhibit the phosphorylation of glycogen synthase kinase 3 (GSK3), thus blocking the transcription of cyclin-dependent kinase (CDK) inhibitor p27 mediated by forkhead box protein O(FOXO), thus promoting the cell cycle process. The inhibition of GSK3 phosphorylation could also prevent the degradation of cyclinD1 (CCND1) stimulated by cell cycle, which in turn increased the expression of CCND1, promoted the transfer of CCND1 to the nucleus, and interfered with the transformation of cell cycle from G1 to S, thus positively regulating cell proliferation [57, 81, 88]. Furthermore, the activated AKT can arbitrarily inhibit Bcl-2-related dead protein (BAD), which leads to the dissociation of Bcl-2 on the mitochondrial membrane. Moreover, the phosphorylation of BAD destroys its ability to bind to Bcl-xl (an antiapoptotic protein), inactivating BAD's ability to induce cell death, and finally inhibiting apoptosis [58, 67]. AKT also promotes the process of cell cycle by phosphorylating and inhibiting cyclin-dependent kinase inhibitors p21 and p27 (p21 and p27 act as G1 checkpoints to prevent cell cycle); AKT also phosphorylate murine double minute 2 (MDM2, a kind of oncoprotein). The phosphorylated MDM2 enters the nucleus, which leads to the degradation of tumor suppressor p53 [77], further inhibits apoptosis, and thus promotes cell proliferation, metabolism, survival, and movement [89, 90]. The induction of matrix metalloproteinase 2 (MMP2) and matrix metalloproteinase 9 (MMP9) by AKT resulted in increased cell migration and invasion [88].

## 4. Conclusion

The PI3K/AKT pathway is abnormally activated in a variety of cancers. As its downstream target, WDHD1 can be phosphorylated by activated AKT. There are three subtypes of AKT, namely, AKT1, AKT2, and AKT3. The three-dimensional structure is very similar. Although it is proved that WDHD1 may be phosphorylated and stabilized by AKT1, thus, in this study, human recombinant AKT1 protein was directly used as a kinase and WDHD1 was used as a substrate, and finally, it is concluded that AKT1 phosphorylates WDHD1 directly [31]. We think that AKT2 and AKT3 are not included in this experiment, so it can not explain that WDHD1 is specifically phosphorylated by AKT1, which has certain limitations. However, it can be confirmed that WDHD1 is indeed a downstream target of PI3K/AKT. Through literature search on various platforms, we found that abnormally high WDHD1 is related to the occurrence and short survival time of tumors. WDHD1 has been identified as a key gene in the occurrence of esophageal cancer [17, 31], but there are few related researches on the mechanism of WDHD1 causing esophageal cancer. By reading relevant literature, we concluded that WDHD1, as the downstream target of the PI3K/AKT pathway, is involved in the occurrence of esophageal cancer. After phosphorylated by AKT, WDHD1 may promote the occurrence of esophageal cancer by regulating cell cycle and inducing DNA replication. We think that WDHD1 may be a tumor factor that plays an irreplaceable role in the occurrence of esophageal cancer. Once the etiology of the patients is determined, it may be a promising therapeutic strategy to selectively target the interaction between the PI3K/AKT pathway and related targets.

## Figures and Tables

**Figure 3:**
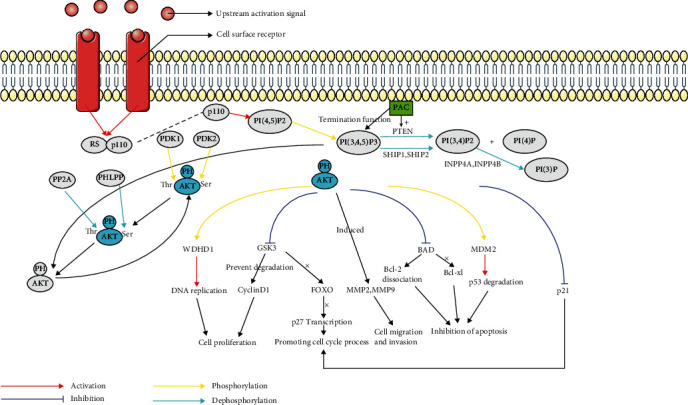
Mechanism of the PI3K/AKT pathway in esophageal cancer. RS: regulatory subunit; AKT (grey): inactive state; AKT (blue): activated state.

**Table 1:** WDHD1 is associated with different tumors.

Tumor	Mechanism	Function
Lung adenocarcinoma	WDHD1 can increase the ubiquitination degradation of microtubule-associated protein RP/EB family member 2 (MAPRE2) [19]	Induces cisplatin resistance [19]
High-risk human papillomavirus- (HPV-) related tumors	1 HPV E7 upregulates the expression of WDHD1 and then GCN5,which activates Akt [20] 2 Downregulation of the WDHD1 gene reduced E7-induced G1 checkpoint abrogation and replication; knockdown of WDHD1 lead to G1 arrest in E7-expressing cells [21]	Promote cell proliferation [20]
PTEN-inactive triple-negative breast cancer	WDHD1 expression is Affected by PTEN-AKT signaling in triple-negative breast cancer(TNBC) cells at both mRNA and protein levels [22]	WDHD1 is an essential gene for the survival of PTEN-inactive TNBC cells [22]
Laryngeal squamous cell carcinoma (LSCC)	WDHD1 and its target gene S-phase kinase-associated protein 2(Skp2) for transcriptional regulation may play a role in the progression of LSCC by regulating the cell cycle [23]	WDHD1 has a potential value in distinguishing LSCC from noncancer [23]
Cholangiocarcinoma (CCA)	miR-494 overexpression promoting CCA cell apoptosis through inhibiting WDHD1 [24]	WDHD1 silencing suppressed tumor formation as well as lymph node metastasis [24]
Non-small-cell lung cancer (NSCLC)	WDHD1 inhibitor induced G2/M phase arrest by regulating the ATM signaling pathway and enhanced irradiation-induced DNA damage by inhibiting the DNA HR repair pathway [25]	WDHD1 inhibition significantly increased the radiosensitivity of NSCLC cells [25]
Pancreatic adenocarcinoma	—	Employs WGCNA to identify WDHD1 is a hub gene for the oncogenic roles during pancreatic adenocarcinoma progression [26]
Cervical cancer	—	WDHD1 is key gene that significantly correlate with cervical cancer lymph node metastasis [27]
Esophageal squamous cell carcinoma (ESCC)	—	Employs WGCNA to identify WDHD1 is a hub gene in ESCC development; WDHD1 expression levels were increased in ESCC tumor tissue [17]
Acute myeloid leukemia (AML)	—	WDHD1 knockdown impaired growth and viability of the cells in primary leukemic cells [28]
Ovarian cancer(OC)	ATR-WDHD1 pathway may play a critical role in platinum resistance of OC [29]	WDHD1 inhibitors can inhibit ovarian tumor growth and overcome platinum drug resistance of OC [29]

WGCNA: weighted gene coexpression network analysis; ATR: ataxia telangiectasia and Rad3-related protein.

**Table 2:** Classes, upstream activation signals, substrates, and functions of PI3K [45–47, 52, 53, 56, 58, 60, 62–66].

PI3K		Catalytic		Regulatory		Upstream activation signal	Substrate	Function
		Gene	Subunit	Gene	Subunit			
Class I	IA	*PIK3CA*	P110*α*	*PIK3R1*	p85*α* P55*α* P50*α*	RTK GPCRs PKC SHP1 Rac Rho Hormonal receptors Src Mutated Ras Various oncogenes Growth factors	PI PIP PIP2	1 Involved in cell metabolism, survival, proliferation, autophagy, growth, chemotaxis, and phagocytosis of huge elements 2 Its pathological overactivity is considered as the driving factor of many cancers, benign diseases and overgrowth 3 PI3K*α* plays an important role in glucose homeostasis, insulin signal transduction, tissue configuration control, and angiogenesis and participates in promoting myocardial growth through the PIP3-dependent pathway 4 PI3K*β* regulates the activity of platelet integrin *α*IIb*β*3 in the context of platelet adhesion and aggregation and plays an important role in platelet function and thrombosis 5 PI3K*δ* signal is structurally activated in many B cell malignant tumors
		*PIK3CB*	p110*β*	*PIK3R2*	p85*β*			
		*PIK3CD*	p110*δ*	*PIK3R3*	p55*γ*			
	IB	*PIK3CG*	p110*γ*	*PIK3R6* *PIK3R5*	p84/p87p101	GPCRs		1 Coordinating immune, inflammatory, and allergic responses, predominantly within hematopoietic cells 2 PI3K*γ*signal is activated in myeloid cells in response to tissue hypoxia
Class II		*PIK3C2A* *PIK3C2B* *PIK3C2G*	C2*α* C2*β* C2*γ*	—	—	Cytokine receptors RTK Integrins	PI PIP2	1 Affecting glucose transport, cell migration, regulation of membrane trafficking, insulin signaling, receptor internalization, and exocytosis 2 Controlling clathrin-mediated endocytosis
Class III		*PIK3C3*	Vps34	*PIK3R4*	Vps15	Glucose starvation Amino acid starvation Insulin stimulation	PI	1 Controlling several membrane trafficking functions, including endosome-lysosome maturation, endosomal protein sorting, autophagy flux, and cytokinesis 2 Involving in growth and survival of cells 3 Regulating intracellular vesicular transport at multiple steps through the production of phosphatidylinositol-3-phosphate

RTK: receptor tyrosine kinases; GPCRs: G-protein-coupled receptors; PKC: protein kinase C; SHP1: Src homology 2 domain-containing protein tyrosine phosphatase 1; PI: phosphatidylinositol; PIP: phosphatidylinositol phosphate; PIP2: phosphatidylinositol-4,5-bisphosphate; PIP3: phosphatidylinositol-3,4,5-triphosphate.
